# Is Pain Time Pattern Predictive of Future Time Perspective in Older Chronic Pain Patients?

**DOI:** 10.1093/geroni/igaa057.1392

**Published:** 2020-12-16

**Authors:** Gillian Fennell, Abby Pui Wang Yip, M Cary Reid, Susan Enguidanos, Elizabeth Zelinski, Corinna Loeckenhoff

**Affiliations:** 1 University of Southern California, Los Angeles, California, United States; 2 Cornell University, Ithaca, New York, United States; 3 Weill Cornell Medical College, New York City, New York, United States

## Abstract

Chronic pain patients constitute 65% of those ages 65 and older in the US. Many affected older adults are challenged to manage physical and psychological consequences tied to the intensity, interference, and temporal pattern of their pain. However, little quantitative research highlights the psychological impact of constant versus intermittent or ever-present-yet-variable pain, even though temporal pain pattern may have meaningful predictive power for wellbeing and future time perspective (FTP). A positive and expansive view of the future is particularly adaptive for this population because it is positively associated with treatment adherence. In this study, we analyzed secondary data to determine whether pain temporal pattern and pain duration are associated with differences in participant scores on Carstensen & Lang’s Future Time Perspective scale. All participants (N=142) were 45 years old and older with non-cancer chronic pain lasting three months or more. There was no significant association between pain time pattern and FTP (p=.35). Additionally, controlling for pain duration, average FTP scores did not vary significantly as a function of time pattern (p=.07). Our analyses demonstrated no significant relationship between pain time pattern and FTP and no significant moderating effect of pain duration. However, in contrast to the previous literature, FTP was not significantly associated with age and negatively (rather than positively) associated with subjective health (r=-.08, p=.35; r=-.24, p<.01), thus raising concerns about the generalizability of these findings. Implications for understanding time perceptions in older pain patients are discussed.

